# The relationship between mindfulness, anxiety and depression during the COVID-19 pandemic: A meta-analysis of correlational studies

**DOI:** 10.3389/fpsyg.2023.994205

**Published:** 2023-02-15

**Authors:** Fuming Xu, Wanling Zhu, Qian Chen, Youmei Tang

**Affiliations:** ^1^Faculty of Education, Yunnan Normal University, Kunming, China; ^2^School of Education Science, Nanning Normal University, Nanning, China

**Keywords:** COVID-19, mindfulness, mental health, anxiety, depression

## Abstract

**Background:**

The emergence of the COVID-19 pandemic has created an environment in which numerous determinants of poor mental health are intensified. Lockdown, re-lockdown, and media coverage of the spread of the virus, have the potential to contribute to increased levels of anxiety and depression. Mindfulness may act as a buffer against COVID-19-related depressive and anxiety disorders.

**Methods:**

We conducted a systematic review and meta-analysis by searching PubMed, PsycINFO, Web of Science, and Google Scholar for any study published between January 2020 and March 2022. In this study, Comprehensive Meta-Analysis Version 3.3 software was applied to evaluate the effect size by random effect model. In addition, the heterogeneity analysis was evaluated using indicators *Q* and *I^2^* indicators. Three methods were used to test for publication bias: funnel plot, Classic Fail-safe N, and Egger’s linear regression. According to the features of the included articles, subgroup analysis was utilized for the moderator analysis of this study.

**Results:**

The analysis finally included 12 articles (16 samples, *N* = 10,940) and obtained 26 independent effect sizes. In accordance with the meta-analysis, in the random effect model, the correlation between mindfulness and anxiety was −0.330 (*p* < 0.001), and the correlation between mindfulness and depression was −0.353 (*p* < 0.001), which supported the effect of mindfulness on anxiety and depression. In the meta-analysis of the correlation between mindfulness and anxiety, study region had an essential moderating effect (*p* < 0.001). The Sample type did not produce a significant moderating effect (*p* = 0.190). The mode of action of mindfulness was a significant moderator (*p* = 0.038). In the meta-analysis of the linkage between mindfulness and depression, regional differences had a significant moderating effect (*p* < 0.001). The sample type had no discernible moderating impact (*p* = 0.213). The mode of action of mindfulness was a significant moderator (*p* = 0.003).

**Conclusion:**

Our meta-analysis indicated that there was an essential correlation between public mindfulness and mental health. Our systematic review added evidence supporting the beneficial nature of mindfulness. A cascading development of beneficial traits that improve mental health may start with mindfulness.

## Introduction

Since the discovery of the first case of new coronary pneumonia in Wuhan, Hubei, China in December 2019, people all over the world have been affected by the COVID-19 pandemic to different degrees. Numerous governments have factored the impact of the COVID-19 epidemic into their policy deliberations. Our daily life is gradually shifting into a new normal brought about by the pandemic. Simultaneously, changes in lifestyle under the new normal, namely, sedentary, prolonged use of electronic devices, changes in eating and resting patterns, etc., can lead to higher levels of anxiety, stress and depression, thus affecting people’s mental health (Huremović, 2019; Arora and Grey, 2020; Rogers et al., 2020; Galli et al., 2022). During the intervals, public health emergencies usually have impacts on individuals and communities such as physical health and mental health. The impacts at the individual level include insecurity, emotional regulation, etc., and the impacts at the community level include economic loss, public places being closed, medical supplies being insufficient, etc. These effects are probable to translate into a range of emotional responses, such as psychological distress, anxiety, and depression (Pfefferbaum and North, 2020). From the outbreak of SARS in 2003, we can observe how quarantine measures affect people’s mental health during public health emergencies. Studies from the time indicated that many people had mental health issues at various levels, including anxiety, depression, panic attacks, and even self-mutilation (Liu et al., 2003). In the long run, isolation measures have adverse effects on people’s risk perception, interpersonal trust and trust in health authorities (Diotaiuti et al., 2021). On the one hand, risk perception affects individual emotional management and mental health (Han et al., 2021); On the other hand, interpersonal trust and trust in health authorities have decreased, which undoubtedly poses a serious challenge to the effectiveness of public management in the new normal.

Studies have demonstrated that levels of anxiety and depression of people increase significantly during the COVID-19 pandemic (Wang et al., 2020), and can reach clinical levels in recovered COVID-19 patients and the general population (Fischer et al., 2020). Studies have also confirmed that older adults are more likely to be socially isolated due to their higher risk of COVID-19-related complications and death (Smith et al., 2020). As an unbiased risk factor for depression, anxiety, and suicide, social isolation has additional negative effects on the elderly’s mental health (WHO, 2020; da Cruz et al., 2022; D’Oliveira et al., 2022). Moreover, among college students, due to the closed management of most schools during the epidemic, factors such as sedentary, unhealthy diet, and restricted physical activity have adversely affected college students’ mental health (Ferrara et al., 2022). Besides, Brooks et al. (2020) presumed that the isolation measures taken in response to the COVID-19 pandemic caused anxiety, depression and other emotional reactions that are not short-lived and situational, but may persist for months or years (Brooks et al., 2020). A variety of research evidence suggests that the effects of isolation on mental health are broad, substantial, and long-term (De Lima et al., 2020; Jin et al., 2021). In conclusion, in the current new normal brought about by the COVID-19 pandemic, people’s mental health problems need to be paid attention to and need urgently to be solved.

Emotional regulation is critical to public mental health, and there have been studies showing that mindfulness is associated with both emotional content and emotion regulation (Didonna, 2021). Mindfulness is typically understood in terms of its process definitions: “non-judgmental awareness of the here and now” and “awareness, the experience of the present moment with acceptance” Another process definition of therapeutic mindfulness is “attentional control,” which refers to refocusing attention on emotional pain management (Didonna, 2021). Bishop et al. (2004) proposed a two-component model of mindfulness, one of which is adopting an approach to one’s experience in the present that is characterized by curiosity, openness, and acceptance. This approach is comparable to the “general tendency of individuals to pay attention to and be aware of the present experience in daily life” measured by the Mindful Attention Awareness Scale (MAAS; Didonna, 2021). A high level of trait mindfulness can therefore delay the emergence of psychopathology, which is how trait mindfulness can be regarded as a psychological resilience component. It could also be described as a risk element wherein a deficiency in the trait of mindfulness raises the possibility of psychopathology (Thompson et al., 2011). We can therefore better understand potential protective or risk factors for mental health by understanding the connection between trait mindfulness and mental health. Additionally, there is growing evidence that individual differences exist in the typical frequency with which people experience states of mindfulness (Mesmer-Magnus et al., 2017). This serves as additional proof that trait mindfulness is a distinct propensity and that each person’s experience of mindfulness training will be unique. Numerous studies found that the negative mental health variables connected to the COVID-19 pandemic, such as anxiety, depression, and concerns about the epidemic, are adversely correlated with mindfulness (Dillard and Meier, 2021).

However, even though previous research has demonstrated that individuals with high levels of trait mindfulness have better responsiveness to emotional stress and better recovery from negative emotions (Fogarty et al., 2013), current research has focused on providing evidence of the effectiveness of mindfulness-based interventions (Kwon and Lee, 2021), few studies have systematically examined the impact of mindfulness as a potential protective factor for the public mental health in response to the COVID-19 pandemic. Therefore, to provide a clearer reference for the public and the government in their response to the COVID-19 epidemic, it is essential to properly review and assess these research results.

This study aimed to provide a systematic review and meta-analysis of published research on the association between mindfulness and mental health since the outbreak of COVID-19. To summarize and evaluate the impact of mindfulness on mental health, we focus on published studies in the context of the COVID-19 pandemic that have examined the relationship between mindfulness and mental health. In accordance with our investigation, most of the current systematic reviews discussed in the context of the COVID-19 pandemic focused primarily on the effects of mindfulness-based interventions. Owing to the fact that we have entered the new normal period of the epidemic, it is crucial to conduct a meta-analysis focusing on the relationship between mindfulness and mental health has become particularly important. Hence, we evaluated the results of the studies included in this meta-analysis, applying the correlation coefficient as the effect size, the region from which the participants came, the type of study sample, and the role of mindfulness in influencing mental health as moderator variables. There is proof that the correlation and structure of mindfulness may vary depending on the sample type. In light of this, we separated the sample types into clinical, community, and college samples (Baer et al., 2006; Tran et al., 2013; Bravo et al., 2018). Similar to this, Chen et al. (2012) noted in their study that there are important distinctions between the effects of meditation on anxiety in Asian and Western nations. As a result, we separated the nations into the regions of Asia, Europe, and North America where the participants were located. In a review of the literature on trait mindfulness, it was discovered that some researchers have frequently employed it as a moderator or mediator in earlier research on the mechanisms impacting mental health and the indirect effects of mindfulness on mental health. Similar to this, several study investigated the major impact of mindfulness on mental health and the influence of mindfulness on mental health when it is the main variable (Karl and Fischer, 2022). Do the outcomes of these two types of activity differ significantly from one another? Few studies on this topic have been done so far. In order to investigate the moderating effects of the studies mentioned, we used the sample type, nation location, and the mode of action of mindfulness as moderating variables. On this basis, we discussed the role of mindfulness on the mental health of the average adult during the COVID-19 pandemic.

## Method

### Inclusion/exclusion criteria

**Figure 1:**
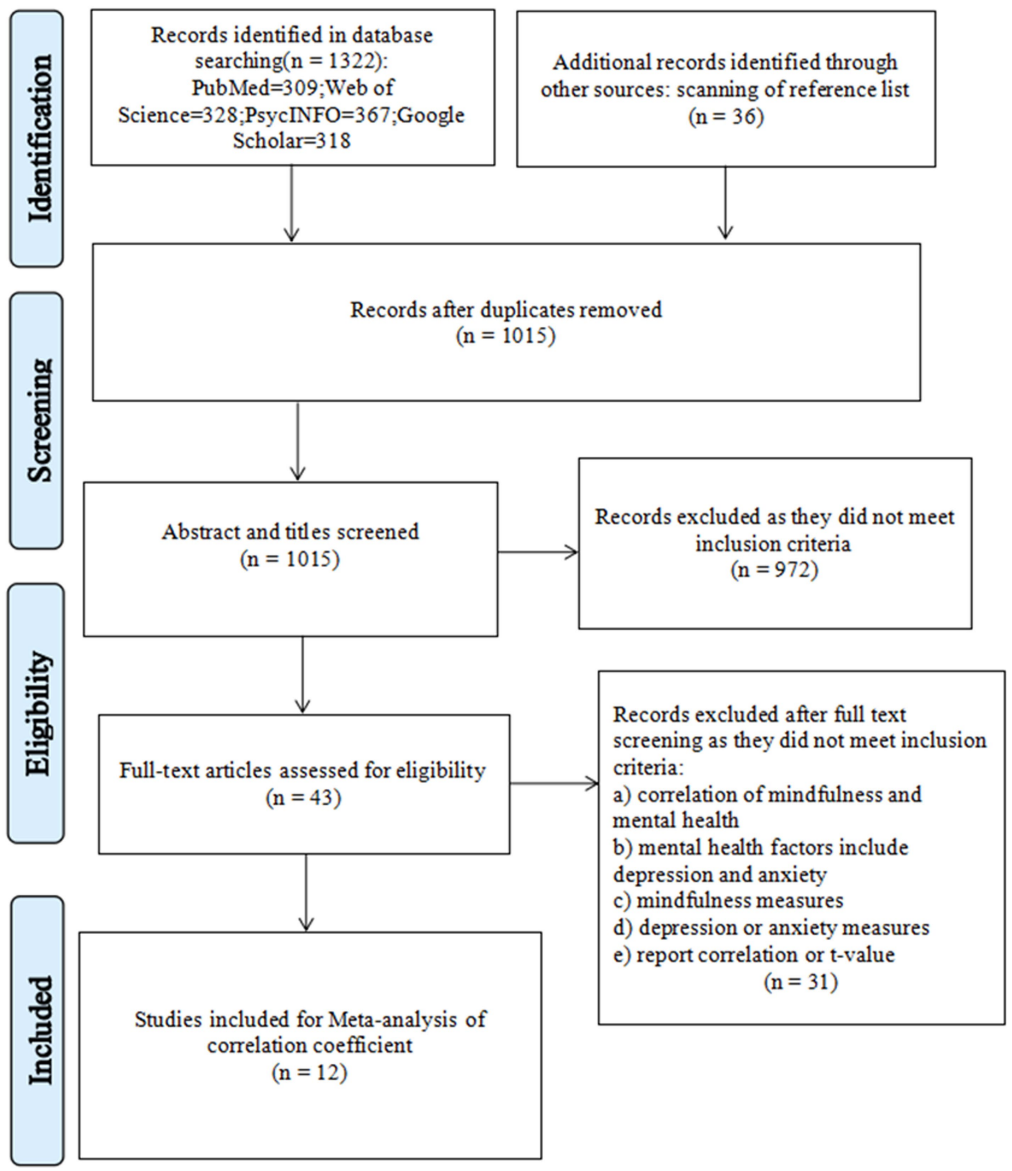
Flow diagram for the search and inclusion criteria.

The included literature conformed to the following criteria: (1) It must be an empirical study examining the correlation between mindfulness and mental health indicators (anxiety, depression, etc.) in the context of the COVID-19 pandemic. (2) The study must have included a scale measuring mindfulness. (3) The study must have included a scale measuring anxiety or depression. (4) All published documents should be in English. (5) The sample size and correlation coefficient, or t-values that can be converted to correlation coefficients, were reported in detail in the text. (6) The included literature should be articles from peer-reviewed journals. Literatures was excluded in accordance with the following criteria: (1) Systematic reviews, meta-analyses, and review literatures was excluded. (2) Studies with only minors were excluded. 0 to 18 years old is the age range for minors. (3) Excluded the studies of mindfulness-based psychological interventions. (4) Excluded conference papers, dissertations and other literature that have not been peer-reviewed. (5) Studies that did not report a Pearson correlation coefficient or explicitly reported a Pearson correlation coefficient were excluded. Figure 1 showed the process of including and excluding literature. A total of 43 articles met the criteria, which enabled them to be further screened for inclusion in the final meta-analysis. The first author and two psychological researchers searched the complete texts of these papers. The meta-analysis ultimately included 12 articles in total.

### Literature search

We conducted a literature search on 1 February 2022, and conducted a systematic literature search in Web of Science, PubMed, PsycINFO, and Google Scholar. The search terms were “mindfulness,” "mental health,” “psychological distress,” and “COVID-19.” We combined the terms “mindfulness*mental health*COVID-19” and “mindfulness*psychological distress*COVID-19” in our search phrase. In addition, some of the literature sources are included as additional sources of information due to the fact that they do not have download rights in PubMed. This part of the literature was obtained through a PubMed search to obtain literature titles and author’s information, and then downloaded from the Taylor & Francis Online and SAGE journals websites. Since the epidemic began in December 2019 and anti-epidemic measures such as lockdown and isolation were implemented in 2020, the period during which the epidemic had a substantial influence on the public began in 2020; therefore, the publication date of the chosen literature is from 2020. The publication dates of the chosen literature range from January 2020 to January 2022, and the date of the final literature supplement is 1 March 2022.

### Study selection

The articles that made it beyond the abstract and title screening step were separately appraised during the full text evaluation phase using the “yes” and “no” codes (Li et al., 2021). Studies that consistently had a code of “yes” underwent final data encoding and information extraction, whereas studies that consistently had a code of “no” were removed. If the two coders’ evaluation results differ, it is up to the first author to decide whether to include the study or not based on the justifications given by the two coders for inclusion or exclusion. The literature selection records of the full-text screening stage were shown in the Supplementary materials.

### Data extraction

For data extraction, the first author first formulated preliminary coding rules as stated in the research purpose and specific circumstances. Subsequently, the five articles included in the analysis were precoded by two coders, and then the first author discussed with the two coders to determine the final coding rules. The last two coders coded all articles in accordance with coding standards. Literature characteristics were categorized into three groups, research characteristics (author, publication year, effective form of mindfulness), participant characteristics (participant nationality, participant occupation, average age of participants, gender ratio), and measurement tools (measurement of mindfulness and mental health factors). By and large, the effect size of each article is coded only once; on the condition that multiple effect sizes are reported in the article, they are coded multiple times on a case-by-case basis. Only two studies from the literature used in this study provided the correlation coefficients for various groups (Dailey et al., 2022; Lam et al., 2022). We entered these values separately because each correlation coefficient operates independently of the others. Two researchers independently extracted information and data from the literature to ensure their accuracy.

### Statistical analysis

This study used Comprehensive Meta-Analysis Version 3.3 (CMA3.3) for the meta-analysis (Borenstein et al., 2009). We used the correlation coefficient *r* as the effect size to explore the pairwise relationship between mindfulness and the indicators of mental health. In general, *r* ≤ 0.1 is considered to be a small effect size, *r* = 0.25 is considered a moderate effect size, and *r* ≥ 0.4 is considered a large effect size (Cohen, 1988). During the extraction process, some literature did not report the correlation coefficient between mindfulness and mental health, but reported *t*-values. Therefore, we used the following formula to convert it into an *r* value (Hunter and Schmidt, 2004; Fritz et al., 2012), and the effect size *r* was calculated as follows:


$$r=\sqrt{\frac{t^{2}}{t^{2}+df}}=\frac{t}{\sqrt{t^{2}+N-2}}$$

If multiple correlation coefficients were reported in the literature, we would input all the correlation coefficients reported in the literature into the software and list them, respectively, in Table 1.

**Table 1:** The characteristics of selected papers.

Article	Study	Mindfulness measures	Anxiety/depression measures	Ethnicity	Region	Sample	Sample type	*N*	Conclusion	*r*
Mann and Walker (2022)	1	Meditative equanimity scale	DASS-21	United States	North America	General population	Community	578	The association between social isolation and psychological distress is indirectly mediated by trait equanimity on the COVID-19.	Anxiety:-0.190
										Depression: −0.030
Vos et al. (2021)	1	TFMQ-SF	DASS-21	Netherlands, Belgium, etc.	Europe	General population	Community	546	Depression, anxiety, and stress were adversely affected by COVID-19 fear, although these effects were attenuated by mindfulness, optimism, and resiliency.	Anxiety: −0.490
										Depression: −0.570
Belen (2022)	1	MAAS	SCL-90-R	Turkey	Europe	Undergraduate students	College	355	The correlation between COVID-19 anxiety and depression is moderated by mindfulness.	Anxiety: −0.490
										Depression: −0.540
Conversano et al. (2020)	1	MAAS	SCL-90	Italy	Europe	General population	Community	6,412	The role of mindfulness in defending against psychological distress brought on by social isolation and quarantining due to COVID-19.	Anxiety: −0.536
										Depression: −0.565
Majeed et al. (2020)	1	MAAS	PHQ-9	Pakistan	Asia	Currently working employees	Community	267	It was discovered that trait mindfulness served as a significant buffer, lowering the unfavorable indirect connection between problematic social media use and depression *via* fear of COVID-19.	Depression: −0.120
Jiménez et al. (2020)	1	SCS-SF-Mindfulness	DASS-21	Spain	Europe	Adult	Community	412	According to the findings, higher levels of Self-kindness, Common Humanity, and mindfulness were associated with not being in treatment, and higher levels of these traits were also associated with better cohabitation.	Anxiety: −0.468
										Depression: −0.552
Chiesi et al. (2022)	1	MAAS	HADS	Italy	Europe	breast cancer patients and survivors	Clinical	409	Personal optimism, hope, self-efficacy, courage, and trait mindfulness served as a protective factor to deal with the stressful condition in COVID-19 pandemic.	Anxiety: −0.350
										Depression: −0.350
Dailey et al. (2022)	1a	MAAS	PROMIS	USA	North America	Sheltering alone	Community	210	The findings highlight the significance of taking social connectedness, mindfulness, and coping into account during a public health crisis.	Anxiety: −0.015
										Depression: −0.001
Dailey et al. (2022)	1b	MAAS	PROMIS	USA	North America	Sheltering with partner only	Community	277		Anxiety: −0.071
										Depression: −0.045
Dailey et al. (2022)	1c	MAAS	PROMIS	USA	North America	Sheltering with children under 18	Community	342		Anxiety: −0.021
										Depression: −0.002
Lam et al. (2022)	1a	CAMS	CES-D	China	Asia	Husband	Community	200	This study made clear the value of investigating the possible advantages of mindfulness not just at the individual level but also at the dyadic level.	Depression: −0.490
Lam et al. (2022)	1b	CAMS	CES-D	China	Asia	Wife	Community	200	Depression: −0.590
Demirdogen et al. (2022)	1	MAAS	DASS-21	Turkey	Europe	Undergraduate students	College	43	Under quarantine conditions, higher levels of depression, anxiety, and stress symptoms were linked to meta-cognition issues and low mindfulness.	Anxiety: −0.360
										Depression: −0.460
Yalçın et al. (2022)	1	MAAS	DASS-21	Turkey	Europe	Undergraduate students	College	506	While COVID-19 fear, depression, anxiety, and stress were adversely correlated with life satisfaction and social support, they were positively correlated with mindfulness and resilience.	Anxiety: −0.40
										Depression:-0.40
Wielgus et al. (2020)	1	FIU-14	STAI	Poland	Europe	General population	Community	170	The development of mental disorders may be mediated by psychological flexibility and mindfulness.	Anxiety: −0.458

The random effects model presupposes that there is not only one true effect size in the meta-analysis, and that it will vary depending on the group of participants in the study and the research tools employed (Borenstein et al., 2009). The regions the participants are from, the variations in how mindfulness affects mental health, etc., have an impact on the relationship between mindfulness and the indicators of mental health examined in this paper. Therefore, this paper used a random-effects model to evaluate the effect size. Additionally, heterogeneity analysis was evaluated using *Q* and *I^2^* indicators. Hunter and Schmidt (2004) pointed out that when *I^2^* > 25%, there is a substantial difference between the included studies, that is, there are potential moderators in the meta-analysis. Previous studies also believed that 25%, 50%, and 75% represent low, medium, and high heterogeneity, correspondingly (Borenstein et al., 2009). When *Q* is significant and *I^2^* ≥ 75%, it shows that there is heterogeneity among studies that cannot be ignored, and it is more prudent to select a random effect model (Huedo-Medina et al., 2006).

Publication bias is that published studies cannot represent of the study population (Kepes et al., 2012). It can affect the validity of meta-analysis results, conclusions and practices based on a meta-analysis (Wei, 2021). Consequently, in the specific meta-analysis process, this paper used three methods: funnel plot, Classic Fail-safe N, and Egger’s linear regression to test publication bias.

## Results

### Search results and study selection

A flowchart of study selection was shown in Figure 1. In the preliminary search, we found a total of 1,358 articles and excluded 343 duplicate articles. In the following, we browsed the title and abstract of each article and excluded 972 articles that did not meet the criteria according to our exclusion criteria. Finally, the first author and two researchers perused the full texts of the initial 43 included papers. 12 papers were ultimately included in our meta-analysis after screening according to the inclusion criteria.

### Study characteristics

This analysis ultimately included 12 articles and obtained 26 independent effect sizes for 10,940 subjects across 16 samples from 12 studies. The samples involved community samples (*k* = 8), university samples (*k* = 3) and clinical samples (*k* = 1). Of the reported mental health indicators, 10 (83.3%) reported the anxiety and 11 (92.0%) reported the depression. In seven of these studies, mindfulness as a mediator or moderator had an indirect effect (58.0%). 64 percent of the articles included the Mindfulness Attention Awareness Scale (MAAS) as a mindfulness measurement instrument. Most of the tools adopted to measure mental health-related indicators were the Depression Anxiety Stress Scale (DASS-21), which accounted for 36%. 82% of participants were from Europe and North America, and 18% were from Asia. The specific studies information was displayed in Table 1.

*Meditative Equanimity Scale*, 20-item Phenomenological Experience of Meditative Equanimity Scale; *TFMQ-SF*, a short version of the Three Facet Mindfulness Questionnaire; *DASS-21*, Depression Anxiety Stress Scale; *MAAS*, Mindful Attention and Awareness Scale; *SCL-90*, The Symptoms Checklist-90; *PHQ-9*, the brief 9-item Patient Health Questionnaire; *SCS-SF-Mindfulness*, Self-Compassion Scale-Short Form; *HADS*, Hospital Anxiety and Depression Scale; *PROMIS*, Patient Reported Outcomes Measurement Information System; *CAMS*, Cognitive and Affective Mindfulness Scale-Revised; *CES-D*, the 10-item Center for Epidemiology Studies Depression Scale; *STAI*, The State–Trait Anxiety Inventory; *FIU-14*, the Freiburg Mindfulness Inventory.

### Publication bias

**Figure 2:**
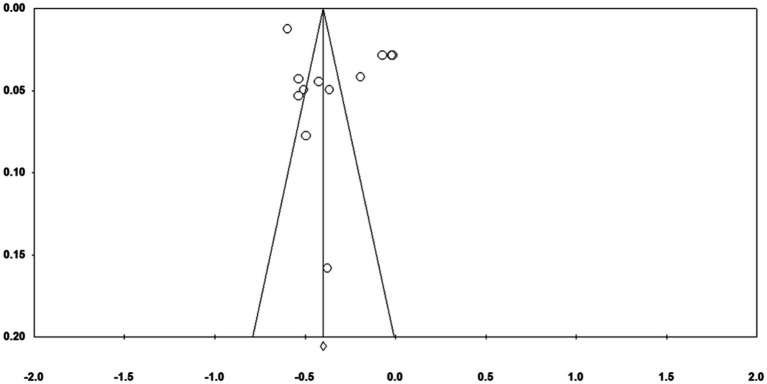
Funnel plot of standard error by Fisher’s Z (anxiety).

**Figure 3:**
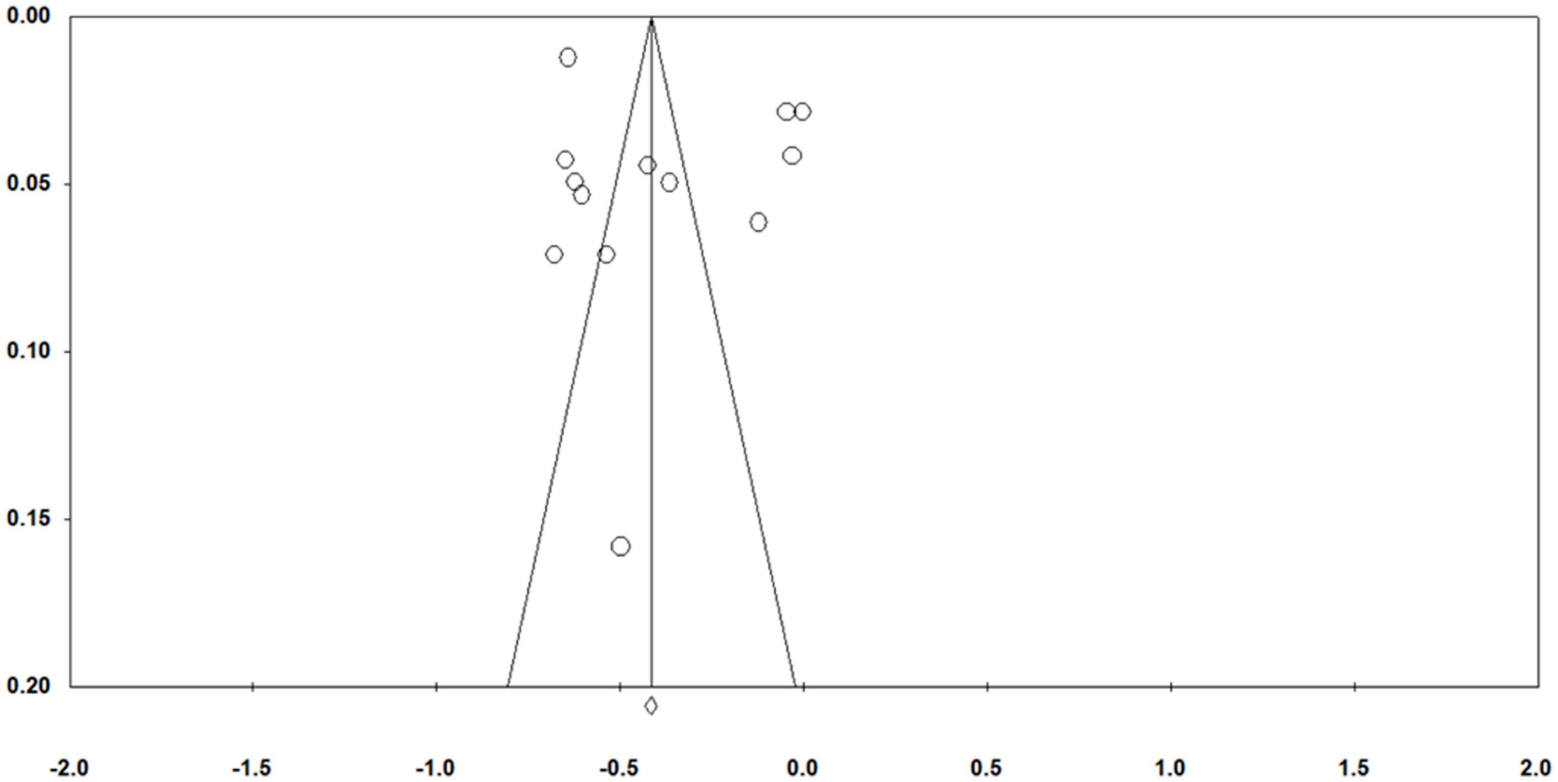
Funnel plot of standard error by Fisher’s Z (depression).

Publication bias can affect the results of the analysis and the validity of the results based on the analysis. Funnel plots are often used for preliminary tests of publication bias. It typically uses Fisher’s Z as the X-axis and the standard error as the Y-axis. From Figures 2, 3 (Figures 2, 3 were anxiety and depression, respectively), most of the effect sizes about mindfulness and anxiety were distributed near the total effect size, the effect sizes of the research findings on mindfulness and depression tended to be evenly distributed on both sides of the total effect size. It suggested that there was no serious publication bias in the research on the relationship between mindfulness and mental health. Since the funnel plot is an intuitive and preliminary test for publication bias, Classic Fail-safe N and Egger’s were further used for more precise tests (see Table 2). The results in Table 2 showed that the Classic Fail-safe N of mindfulness and anxiety was 3,416, that was, an additional 3,416 research papers were needed to overturn the results of this analysis; the Classic Fail-safe N of mindfulness and depression was 4,499, that was, an additional 4,499 research papers were needed to overturn the analysis results of this analysis. It indicated that in this meta-analysis, there was no significant publication bias. At the same time, the *p*-values in Egger’s test were 0.27 and 0.31, both of which were greater than 0.05, indicating that there was no publication bias in this meta-analysis.

**Table 2:** Publication bias of analysis.

	Classic fail-safe *N*	Egger’s intercept	SE	LL	UL	*p*-value
Anxiety	3,416	5.20	4.46	−4.73	15.13	0.27
Depression	4,499	4.80	4.46	−4.95	14.50	0.31

### Overall model

**Figure 4:**
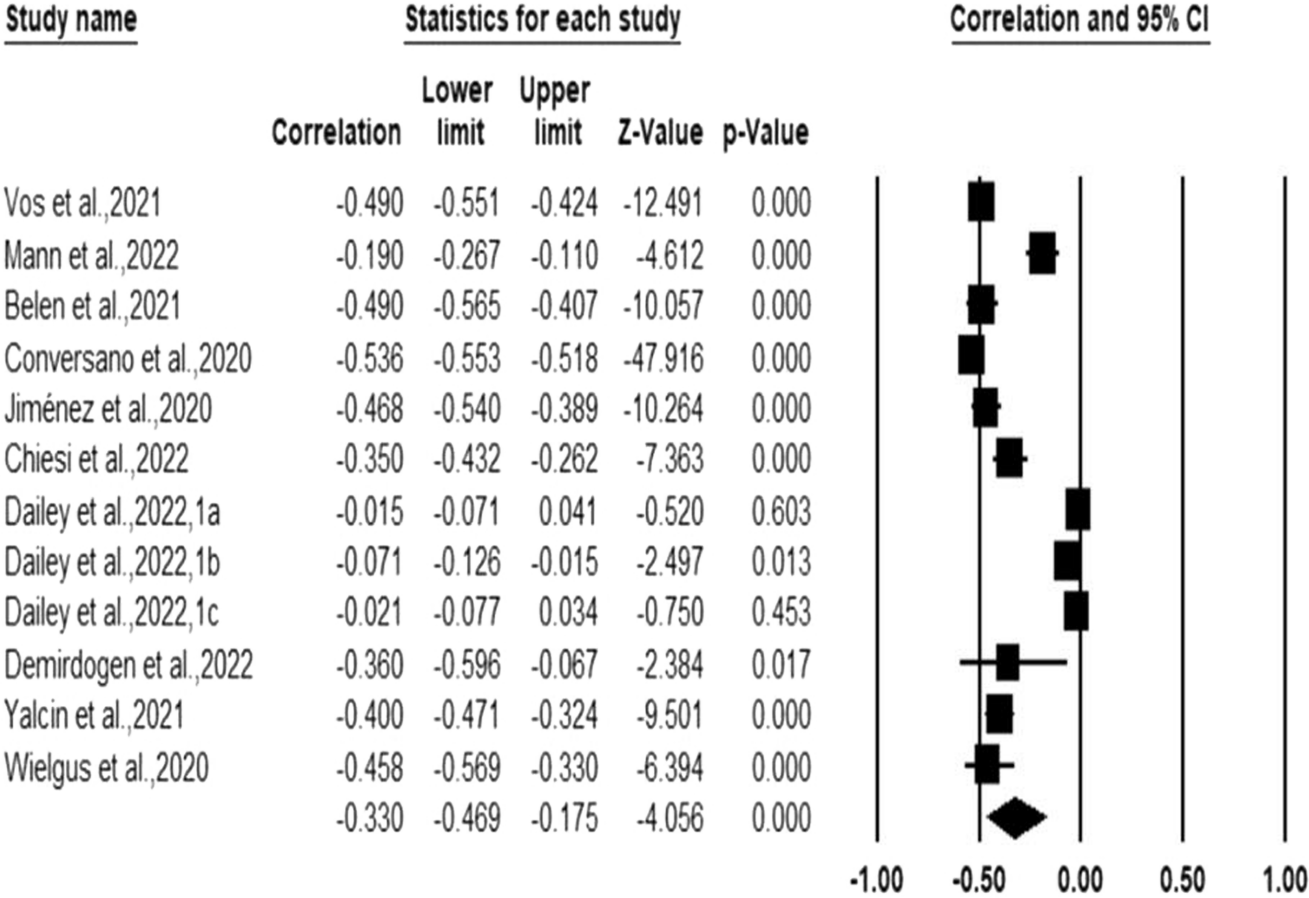
Forest plot (anxiety).

**Figure 5:**
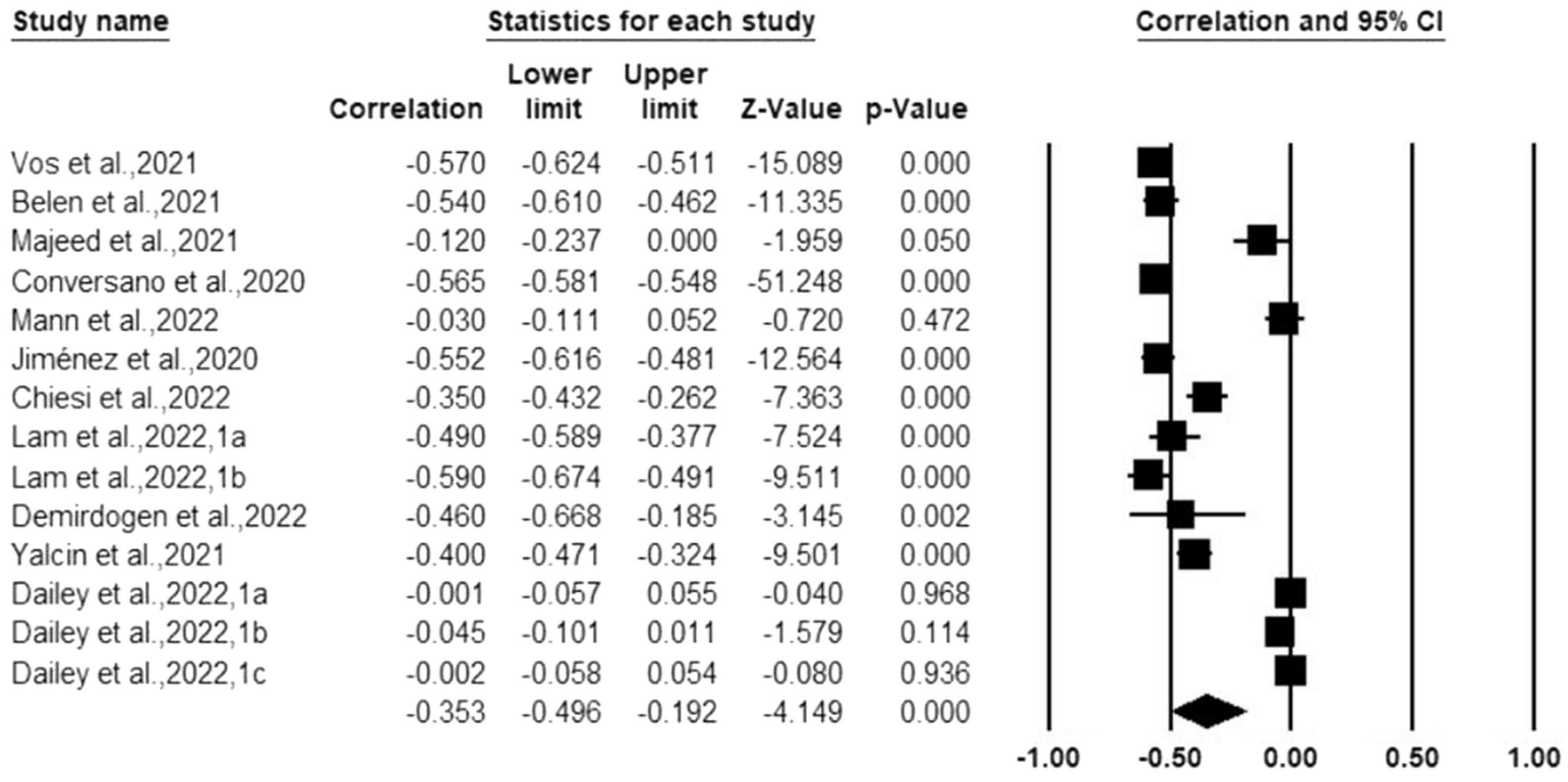
Forest plot (depression).

As shown in Table 3, in the random effect model, the correlation between mindfulness and anxiety was −0.330 (95%CI: −0.469 ~ −0.175, *p* < 0.001), and the correlation between mindfulness and depression were −0.353 (95%CI: −0.496~−0.192, *p* < 0.001), which supported the effect of mindfulness on anxiety and depression. All were medium effect sizes. Figures 4, 5 display the effect sizes of the various anxiety and depression studies, respectively.

**Table 3:** Overall model.

	*k*	*N*	95%CI				
			*r*	LL	UL	*Z*-value	*p*-value
Anxiety	12	10,273	−0.330	−0.469	−0.175	−4.056	0.000
Depression	14	10,940	−0.353	−0.496	−0.192	−4.149	0.000

### Heterogeneity analysis

From Table 4, we could see that in the correlation analysis of mindfulness and anxiety, *I^2^* = 98.612, *Q* = 792.759, *df* = 11, *p* < 0.001, indicating that there was a high heterogeneity among the 12 research results of mindfulness and anxiety. The correlational analysis of mindfulness and depression, *I^2^* = 98.817, *Q* = 1098.677, *df* = 13, *p* < 0.001, indicating that there was also high heterogeneity among the 14 research results of mindfulness and depression. Due to the high heterogeneity among the results of the included studies, we examined the moderators in terms of regions, sample types and the effective form of mindfulness.

**Table 4:** Heterogeneity analysis.

	*Q*-value	*df*(*Q*)	*p*-value	*I*-squared	Tau squared
Anxiety	792.759	11	0.000	98.612	0.082
Depression	1098.677	13	0.000	98.817	0.107

### Moderator analysis

To investigate the moderating effect of mindfulness on mental health, we used random-effects models to examine the moderating effects of the participant’s region, sample type, and the effective form of the mindfulness. The consequences were reported in Table 5. In the meta-analysis of the correlation between mindfulness and anxiety, regional differences had a remarkable moderating effect on the correlation coefficient between mindfulness and anxiety (*p* < 0.001), Europe (−0.457, 95%CI: −0.512~−0.398; *k* = 8) compared with North America (−0.070, 95%CI: −0.136 ~ −0.002; *k* = 4) produced a larger effect size; Sample type did not produce a significant moderating effect on the correlation coefficient between mindfulness and anxiety (*p* = 0.190); mindfulness’s effect form was a significant moderator, modifying the correlation coefficient between mindfulness and anxiety (*p* = 0.038), when mindfulness was the main variable affecting anxiety level, its effect size was larger (−0.445, 95%CI: −0.537~−0.343;*k* = 4). When moderator variables influenced anxiety indirectly, their effect size was modest. (−0.265, 95%CI: −0.402~−0.116; *k* = 8).

**Table 5:** Moderation analysis.

				95%CI		Within-group heterogeneity			Tests for subgroup difference (random effect)		
	*k*	*N*	*r*	Lower	Upper	*Q*-value	*p*-value	*I*-square	*Q*-value	*df*	*p*-value
Anxiety											
Region									71.069	1	0.000
North America	4	1820	−0.070	−0.136	−0.002	14.529	0.002	79.352			
Europe	8	8,853	−0.457	−0.512	−0.398	37.758	0.000	81.461			
Sample type									3.321	2	0.190
College	3	904	−0.436	−0.505	−0.362	2.948	0.229	32.147			
Clinical	1	409	−0.350	−0.432	−0.262	0	1.000	0.000			
Community	8	9,360	−0.294	−0.480	−0.083	785.189	0.000	99.108			
The mode of action									4.285	1	0.038
Indirect	8	2,925	−0.265	−0.402	−0.116	206.757	0.000	96.614			
Main	4	7,739	−0.445	−0.537	−0.343	34.574	0.000	91.323			
Depression											
Region									127.338	2	0.000
North America	4	1,820	−0.018	−0.048	0.012	1.634	0.652	0.000			
Europe	7	8,683	−0.500	−0.564	−0.431	48.871	0.000	87.723			
Asia	3	667	−0.416	−0.655	−0.102	39.356	0.000	94.918			
Sample type									3.094	2	0.213
College	3	904	−0.469	−0.573	−0.351	6.747	0.034	70.359			
Clinical	1	409	−0.350	−0.432	−0.262	0.000	1.000	0.000			
Community	10	9,857	−0.319	−0.503	−0.108	1084.646	0.000	99.170			
The mode of action									8.593	1	0.003
Indirect	8	3,022	−0.229	−0.389	−0.055	285.054	0.000	97.544			
Direct	6	8,139	−0.495	−0.571	−0.411	49.604	0.000	89.920			

Regional distinctions had a significant moderating effect on the correlation coefficient between mindfulness and depression (*p* < 0.001) in the meta-analysis of the correlation between mindfulness and depression. Europe (−0.500, 95%CI: −0.564~−0.431; *k* = 7) produced the largest effect size, followed by Asia (−0.416, 95%CI: −0.655~0.102; *k* = 3), North America (−0.018, 95%CI: −0.048~0.012; *k* = 4); the sample type did not produce a crucial moderating effect on the correlation coefficient between mindfulness and depression (*p* = 0.213); The effective form of the mindfulness was a substantial moderator, moderating the correlation coefficient between mindfulness and depression (*p* = 0.003), when mindfulness was the main variable affecting the level of depression, its effect size was larger (−0.495, 95%CI: −0.571~−0.411; *k* = 6). When the variable affected anxiety level indirectly, its effect size was small (−0.229, 95%CI: −0.389~−0.055; *k* = 8).

## Discussion

According to the findings of our meta-analysis, a person’s level of mindfulness may be a safeguard for their mental health. In the Covid-19 pandemic environment, mindfulness influences how people react to the detrimental effects of lockdowns and isolation. From another perspective, mindfulness may be used as a buffer to alleviate negative emotions in particular depression and anxiety brought about by the COVID-19 epidemic, thereby protecting individual mental health. Furthermore, in different sample groups (community, college, clinical), the effect sizes of mindfulness and mental health have reached a moderate level or above, indicating that mindfulness as a protective factor for mental health is universal. Consequently, the counselor should promote the client’s level of mindfulness in future work and assist the client in surviving epidemic-related adversity more effectively to improve the client’s mental health (Dailey et al., 2022).

But there are still some aspects of the literature we included that demand our attention. The majority of the scales used to measure mindfulness in the included literature are the Mindful Attention Awareness Scale (MAAS), whereas Mann and Walker (2022) employed the 20-item Phenomenological Experience of Meditative Equanimity Scale. They did not employ the definition used by the majority of studies, as was indicated in the introduction of their literature. Based on the study, mindfulness involves equanimity. It is described as being receptive to and accepting of all events (Desbordes et al., 2014; Mann and Walker, 2022). Instead, equanimity is thought to be a component of mindfulness, as per historical meditation teachings theories (Zeng et al., 2015; Mann and Walker, 2022). According to the Buddhist meditation tradition, equanimity is considered to be a key outcome of meditation practices (Desbordes et al., 2014). This may be the reason why this study’s findings on the relationship between mindfulness and mental health were less significant than those of most other research. This may suggest that various aspects of current mindfulness have various consequences on mental health.

In addition, the Daily et al. trial also had a small impact size, as we can clearly see. The literature has also revealed that this outcome is related to racial minorities’ lower reporting and awareness rates of distress symptoms (Liu et al., 2021; Dailey et al., 2022). Dailey et al. (2022) pointed out that racial minorities’ social connections and economic status may have an impact on their level of mindfulness and mental health. Our analysis covered two literatures from North American. 61.3% of participants were white and 18.5% were African Americans, according to Dailey et al. (2022). According to Mann and Walker (2022), 72% of their participants were white and 11.2% of their participants were African Americans. Small effect sizes were reported in both experiments (see Table 1 for details). We also looked at the literature from Europe. The ethnicity of the participants was only mentioned in one study. According to the study, Spain makes up 91% of the total, while Mexico and Peru combined make up 4.6% (Jiménez et al., 2020). Because there is less literature from the European region reporting on the percentage of ethnic minorities. The evidence at hand does not allow us to make any firm inferences. However, we may make some educated guesses based on the evidence that is currently available and two studies from North America. We infer from this that probably minorities report less psychological distress symptoms. Further, we hypothesize that racial disparities might function as a moderating factor in the meta-analysis of mental health. However, only a tiny number of studies have discussed participant ethnicity. This important factor is rarely discussed in other meta-analysis literature (Chu and Mak, 2019; Fischer et al., 2020). To demonstrate the reliability of this variable, more research is required.

Only Chiesi et al. (2022) examined clinical samples in our analysis, and those were breast cancer patients and survivors. Even though the study found a strong link between mindfulness and psychological distress, our analysis found only a moderate correlation. However, growing evidence throughout the pandemic indicates that cancer patients are more likely than the general population to have COVID-19-related symptoms (Baker, 2020; Chiesi et al., 2022). To show that mindfulness can operate as a protective factor in lowering anxiety and depression during clinically significant health safety events like the COVID-19 pandemic, more samples must be examined. In a similar vein, research have revealed that women are more susceptible to anxiety and depression in times of pandemics or clinical diagnosis (Rosenfield and Mouzon, 2013). However, gender is not appropriate for moderating analysis in this analysis due to the minimal number of our effect sizes (Viechtbauer et al., 2015; Cheung and Vijayakumar, 2016). In order to investigate whether gender can moderate the association between mindfulness and mental health, more study is required.

According to studies, people who are mindful are more likely to find other mindful people to date (Garcia et al., 2014; Lam et al., 2022). Intimate relationships’ effects on mindfulness and mental health were studied in two of the research in our study (Dailey et al., 2022; Lam et al., 2022). Its results suggest that the benefits of mindfulness may not be wholly personal but rather have a binary impact (Lam et al., 2022). This implies that the association between mindfulness and mental health may be influenced by close relationships. This could be a beneficial aspect to take into account in the upcoming research on the effects of isolation on mental health.

In this systematic review and meta-analysis, two moderators significantly moderated the association between mindfulness and mental health. The first is the region where the participants come from. The correlation between mindfulness and mental health was highest in Europe, followed by Asia, and lowest in North America, according to the results of an analysis. Based on previous research, we believe this result may be the result of two influential factors. First, the literature that has been included has some limitations. In the North American literature we analyzed, there may be a trend for minorities to report fewer distressing symptoms. Additionally, the concept of mindfulness used in the study by Mann et al. differs significantly from definitions found in other works of literature. The second is cross-culture differences. People in different countries have different responses to stress and trauma (Olff et al., 2021), so people’s mental health under the COVID-19 epidemic are not the same, and the effect of mindfulness on mental health problems is also different. In addition, the Asian sample included in this study is relatively small, but China, as a country that discovered the first confirmed case of new coronary pneumonia and strictly implemented prevention and control policies, research on the impact of isolation, the importance of blockade, and other measures on mental health and the role of mindfulness cannot be overstated, future research could focus on the role of mindfulness in mental health in China.

In addition to the regional moderator, the mode of action of mindfulness also significantly moderated the relationship between mindfulness and mental health. In the study, we believe that the direct effect of mindfulness on mental health is the predominant effect, and the effect of mindfulness as a mediator and moderator on mental health is called an indirect effect (Wei, 2021). Compared with the indirect effect, the direct effect of mindfulness has a higher correlation with mental health, indicating that mindfulness as a major factor significantly alleviates anxiety and depression. But when it is combined with other protective factors (such as social connection) to affect anxiety and depression, the alleviating effect of mindfulness is relatively low. And it provides us with a new implication. During the epidemic, when mindfulness is a state-like trait, does it play a more important role in alleviating mental health problems? This deserves further investigation.

## Conclusions, limitations, and future research

Our study provides some evidence for mindfulness as a protective factor for mental health. For workers engaged in mental health interventions, we suggest that counselors need to consciously identify and improve the level of mindfulness of their clients in order to more efficiently help clients through pandemic-related adversity and improve their own mental health. For school and community managers, we suggest that in follow-up management, individuals with low mindfulness and high stress should be thoroughly screened, and interventions should be made for public mental health problems in order to improve the efficiency of public management.

Nonetheless, the included literature for this analysis has certain limitations. The first is that most of the tools used to measure mindfulness are the Mindfulness Attention Awareness Scale (MAAS). Although MAAS has high internal consistency in samples of college students and the general population, its one-factor structure makes research on mindfulness relatively single, and MAAS emphasizes an aspect that is negatively correlated with dissociative symptoms and absent-mindedness (Didonna, 2021). When using it to measure mindfulness, there may be a certain bias and it is not comprehensive enough. Therefore, in future research, other scales of mindfulness, such as the Five-Factor Mindfulness Questionnaire (FFMQ), should be used to obtain deeper and more comprehensive results in the meta-analysis by comparing different measurement tools.

Second, the clinical samples included in this analysis were small, and the university samples were all from Turkey. As a result, the heterogeneity of clinical samples cannot be calculated, and the heterogeneity of university samples is low. Therefore, results for sample types need to be interpreted with caution.

Third, the research is basically from regions with relatively developed economies. For instance, there are few studies in Africa, and some studies have shown that it is unfair to deal with the epidemic. Namely, low-income countries do not have enough vaccines, which will affect people’s response to the COVID-19 epidemic. Therefore, research on mental health and the role of mindfulness in impoverished regions or countries’ future care must be given more consideration (Olff et al., 2021).

Then, Anxiety and depression were the indices of mental health that we used. However, number of studies have used measures of positive mental health, such as resiliency and hope (Vos et al., 2021; Chiesi et al., 2022). There were insufficient data to do a meta-analysis, despite the fact that some of the literature we included also mentioned positive mental health indicators. As a result, later systematic reviews and meta-analyses might concentrate on the influence of mindfulness on indices of positive mental health.

Furthermore, results for both depression and anxiety are reported in the majority of studies on detrimental indicators of mental health. The reported results might be correlated because the study used the same researchers and subjects. It is common practice to discuss anxiety and depression separately in the majority of recent meta-analyses of mental health, including our work (Torquati et al., 2019; Fischer et al., 2020; Serrano-Ripoll et al., 2020). Potential correlations between variables may be missed in this kind of study (Wei, 2021). The use of SEM-based meta-analysis in future studies to investigate the connection between mindfulness and mental health is thus a possibility.

Finally, due of the pandemic, the literature used in our meta-analysis was gathered using an online survey. And the majority of measurement devices use a self-report scale. However, participants’ subjective perceptions, symptom minimizing, and ambiguity in item interpretation may have an impact on the self-report scale (Bergomi et al., 2013; Dailey et al., 2022). The generalization and application of the findings of this investigation should therefore be carefully considered.

In summary, the available evidence found that mindfulness affects how individuals respond to the negative impacts of lockdowns and quarantines during the COVID-19 pandemic. From another angle, using mindfulness as a safeguard can help people cope with the distress brought on by the COVID-19 pandemic. Individuals in various regions, however, reported considerably varying levels of mindfulness and mental health due to cultural and racial disparities. Counselors and community leaders should assess each person’s level of mindfulness in order to provide mental health improvement support. Special consideration should be given to the mental health of local ethnic minorities and lone residents, and timely psychological intervention should be given to these populations.

## Data availability statement

The original contributions presented in the study are included in the article/Supplementary material, further inquiries can be directed to the corresponding authors.

## Author contributions

FX and WZ designed and performed the research and analyzed and wrote up the research. QC and YT critically reviewed and edited the manuscript. All authors contributed to the article and approved the submitted version.

## Funding

This study was supported by the National Natural Science Foundation of China (grant nos. 72164028 and 71971103).

## Conflict of interest

The authors declare that the research was conducted in the absence of any commercial or financial relationships that could be construed as a potential conflict of interest.

## Publisher’s note

All claims expressed in this article are solely those of the authors and do not necessarily represent those of their affiliated organizations, or those of the publisher, the editors and the reviewers. Any product that may be evaluated in this article, or claim that may be made by its manufacturer, is not guaranteed or endorsed by the publisher.
